# Cell autonomous role of border associated macrophages in ApoE4 neurovascular dysfunction and susceptibility to white matter injury

**DOI:** 10.21203/rs.3.rs-3222611/v1

**Published:** 2023-08-04

**Authors:** Costantino Iadecola, Antoine Anfray, Samantha Schaeffer, Yorito Hattori, Monica Santisteban, Nicole Casey, Gang Wang, Michael Strickland, Ping Zhou, David Holtzman, Josef Anrather, Laibaik Park

**Affiliations:** Weill Cornell Medicine; Weill Cornell Medicine; Weill Cornell Medicine; National Cerebral and Cardiovascular Center; Weill Cornell Medicine; Weill Cornell Medicine; Feil Family Brain and Mind Research Institute - Weill Cornell Medicine; Washington University in St. Louis; Weill Cornell Medical College; Washington University in St. Louis; Feil Family Brain and Mind Research Institute; Feil Family Brain and Mind Research Institute, Weill Cornell Medicine, New York, NY

## Abstract

Apolipoprotein-E4 (ApoE4), the strongest genetic risk factor for sporadic Alzheimer’s disease, is also a risk factor for microvascular pathologies leading to cognitive impairment, particularly subcortical white matter injury. These effects have been attributed to alterations in the regulation of the brain blood supply, but the cellular source of ApoE4 and the underlying mechanisms remain unclear. In mice expressing human ApoE3 or ApoE4 we report that border associated macrophages (BAM), myeloid cells closely apposed to neocortical microvessels, are both the source and the target of the ApoE4 mediating the neurovascular dysfunction through reactive oxygen species. ApoE4 in BAM is solely responsible for the increased susceptibility to oligemic white matter damage in ApoE4 mice and is sufficient to enhance damage in ApoE3 mice. The data unveil a new aspect of BAM pathobiology and highlight a previously unrecognized cell autonomous role of BAM in the neurovascular dysfunction of ApoE4 with potential therapeutic implications.

## Introduction

The integrity of the neurovasculature is critically important for cognitive health, and neurovascular dysfunction and damage have been implicated in a wide variety of conditions associated with cognitive impairment^1, 2^. In particular, microvascular damage to the subcortical white matter is a major contributor to age-related dementia ^3, 4^ and may also play a role in Alzheimer’s disease (AD) ^5–8^. Supplied by terminal arterioles with limited collateral flow from adjacent vascular territories ^9, 10^, the subcortical white matter is particularly vulnerable to microvascular injury ^11, 12^. While vascular risk factors, such as hypertension and diabetes, are strongly linked to white matter lesions, accumulating evidence indicates that ApoE4, the leading genetic risk factor in sporadic AD, also increases the risk for cognitive impairment produced by vascular factors ^13–15^. Thus, ApoE4 carriers exhibit vascular pathology, microvascular alterations, and more white matter lesions linked to cognitive impairment^16–22^. Furthermore, in Aβ immunotherapy ApoE4 positivity increases the incidence of amyloid related imaging abnormalities (ARIA), a treatment-limiting adverse event related to cerebral amyloid angiopathy and microvascular dysfunction ^23–25^, highlighting the negative neurovascular impact of ApoE4. Thus, ApoE4 promotes white matter injury as well as other neurovascular pathologies.

ApoE4 is well-known to be associated with alterations of the neurovasculature. ApoE4-positive individuals have dysregulated cerebral blood flow (CBF) ^26–30^, as well as increased permeability of the blood brain barrier (BBB) in the setting of AD ^31, 32^. Mice expressing human ApoE4 under the control of the mouse ApoE promoter (ApoE4-TR mice) exhibit a profound disruption in the ability of neural activity to increase cerebral blood flow (CBF) and of cerebral endothelial cells to regulate microvascular perfusion ^33^, the integrity which is required for normal cognition ^34, 35^. In a model of white matter injury produced by bilateral common carotid artery stenosis (BCAS) ApoE4-induced neurovascular dysfunction reduces white matter CBF and enhances white matter lesions and cognitive deficits ^33^. These alterations occur at an early age (2–3 months) and precede the BBB dysfunction ^33, 36^. However, the cellular source(s) of ApoE4, the effector cells in the cerebral microvasculature and the signaling mechanisms driving the dysfunction remain unclear.

The brain harbors myeloid cells that arise from the yolk sac early in development and populate different sites in the brain and its coverings in the post-natal period ^37^. While microglial cells reside in the brain parenchyma, border associated macrophages (BAM) seed the meninges, the choroid plexus, and the space surrounding microvessels as they dive into the brain (perivascular space) ^38, 39^. BAM are enriched with free radical producing enzymes and, owing to their proximity to pial arterioles in the leptomeninges and to penetrating arterioles in the perivascular space, have recently emerged as a major cause of neurovascular dysfunction in models of hypertension, vascular amyloid pathology, and sub-arachnoid hemorrhage ^40–44^.

In the present study we investigated the sources and targets of ApoE responsible for neurovascular dysfunction and the mechanisms of the effect. We found that ApoE4 acts on BAM to alter critical cerebrovascular regulatory mechanisms through NADPH oxidase dependent production of reactive oxygen species (ROS). The dysfunction is abolished by BAM depletion or by genetic deletion of ApoE4 selectively in BAM, indicating that these cells are the sole source of the ApoE4 mediating the deleterious vascular effects. Using a bone marrow transplantation strategy, we found that ApoE4-positive BAM induce neurovascular dysfunction in ApoE3-TR mice, whereas ApoE3-positive BAM rescue neurovascular dysfunction in ApoE4-TR mice, indicating that BAM are also the target of ApoE4. Attesting to the pathogenic effect of BAM ApoE4 on white matter injury, ApoE4-positive BAM enhance white matter damage and cognitive impairment in ApoE3-TR mice, while ApoE3-positive BAM rescue this phenotype in ApoE4-TR mice. The findings establish BAM as both the source and the target of the ApoE4 acting on the cerebral microvasculature, unveiling a previously unappreciated cell autonomous role of brain-resident macrophages in the neurovascular dysfunction and propensity of white matter injury associated with ApoE4.

## Results

### 1. Neocortical application of ApoE4, but not ApoE3, attenuates neurovascular responses initiated by neural activity and the endothelium

**Figure 1:**
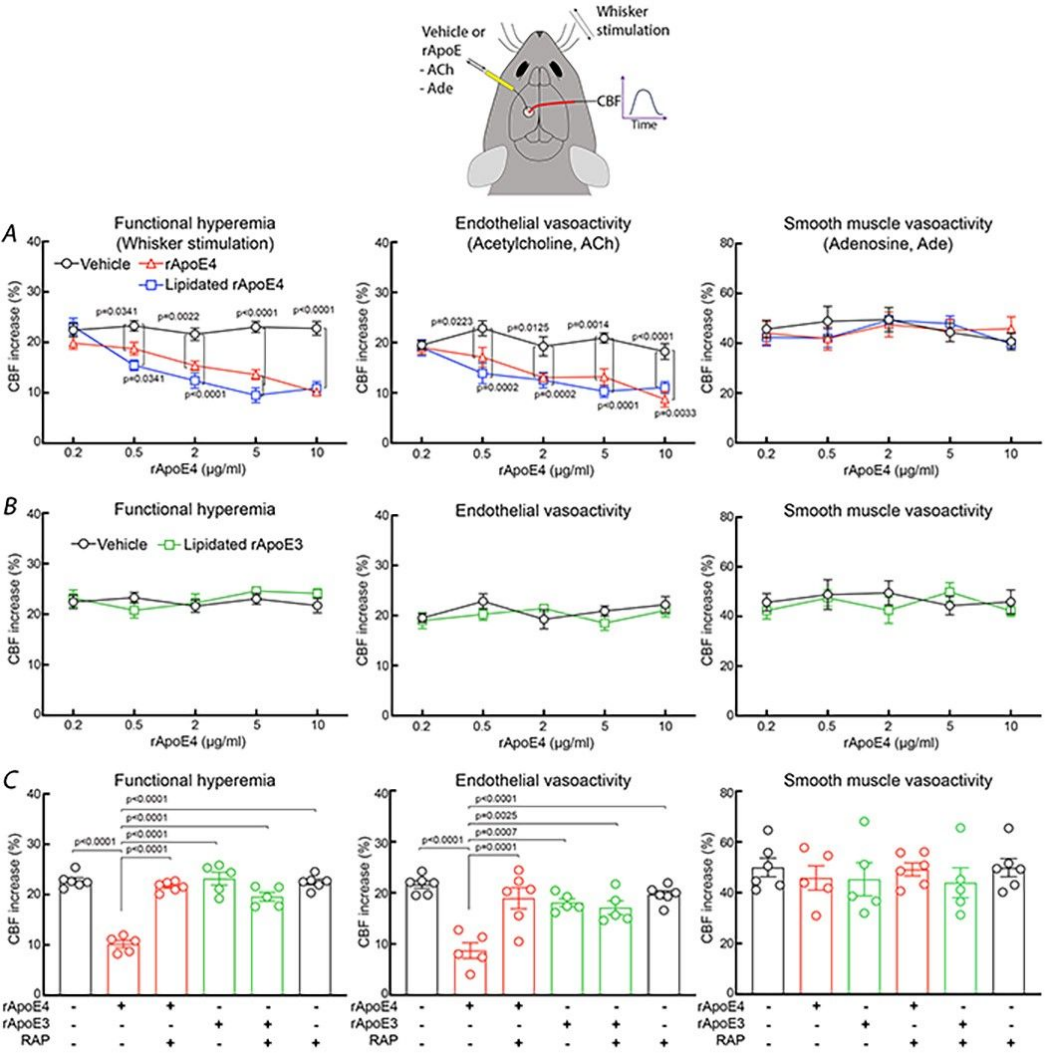
Recombinant ApoE4 (rApoE4) or lipidated rApoE4 alters functional hyperemia and endothelial vasoactivity. ***A.*** Neocortical superfusion of recombinant ApoE4 (rApoE4) or lipidated rApoE4 attenuates functional hyperemia produced by whisker stimulation and the increase of CBF produced by neocortical superfusion of the endothelium-dependent vasodilator acetylcholine. The increase in CBF produced by neocortical superfusion of the smooth muscle relaxant adenosine (smooth muscle vasoreactivity), is not impaired. ***B***. Neocortical superfusion of lipidated rApoE3 does not affect functional hyperemia, endothelial or smooth muscle vasoactivity. ***C***. Neocortical pretreatment with the ApoE receptors inhibitor receptor-associated protein (RAP) (200nM) prevents rApoE4 from altering functional hyperemia and endothelial vasoactivity but does not affect smooth muscle vasoreactivity. N=5/group; one-way ANOVA with Tukey’s test; data presented as mean±SEM.

ApoE4-TR mice exhibit profound alterations in the increases in CBF induced by neural activity and by vasodilating agents acting through the endothelium ^33^. To determine if ApoE4 is directly responsible for these neurovascular effects we examined if topical application of recombinant (r) ApoE4 to the neocortex reproduces the neurovascular dysfunction observed in ApoE4-TR mice ^33^. CBF was monitored in the whisker barrel cortex by laser-Doppler flowmetry in anesthetized WT mice (age 3–4 months) with controlled blood pressure and blood gasses ^45^. Bathing the exposed neocortex with rApoE4 (0.2–10 μg/ml) attenuated the increase in CBF induced by mechanical stimulation of the facial whisker in a concentration related manner (Fig. 1A). Similarly, rApoE4 attenuated the rise in CBF induced by neocortical application of acetylcholine, a vasodilator that acts by releasing nitric oxide from the cerebral endothelium (Fig. 1A) ^46^. The CBF response to application of the smooth muscle relaxant adenosine was not attenuated (Fig. 1A), attesting to the selectivity of the effect of ApoE4 on functional hyperemia and endothelial vasoactivity. Lipidation influences ApoE biological activity ^47, 48^, but the neurovascular effects of lipidated rApoE3 and rApoE4 were comparable to those of the non-lipidated ApoE (Fig. 1A). The reduction of functional hyperemia tended to be more marked with lipidated rApoE4 (Fig. 1A), possibly due to lipidation enabling deeper penetration into the whisker barrel cortex. rApoE3 was devoid of cerebrovascular effects (Fig. 1B). To test if the neurovascular effects of rApoE4 were receptor mediated we applied rApoE4 after pre-treatment of the neocortex with receptor associated protein (RAP), an inhibitor of ApoE receptors ^49^. We found that RAP by itself did not affect cerebrovascular responses, but completely reversed the rApoE4-induced dysfunction (Fig. 1C). These observations indicate that exogenous ApoE4 reproduces the neurovascular dysfunction observed in ApoE4-TR mice and that the effect is receptor mediated.

### 2. ApoE4 induces neurovascular dysfunction through ROS production in BAM

**Figure 2:**
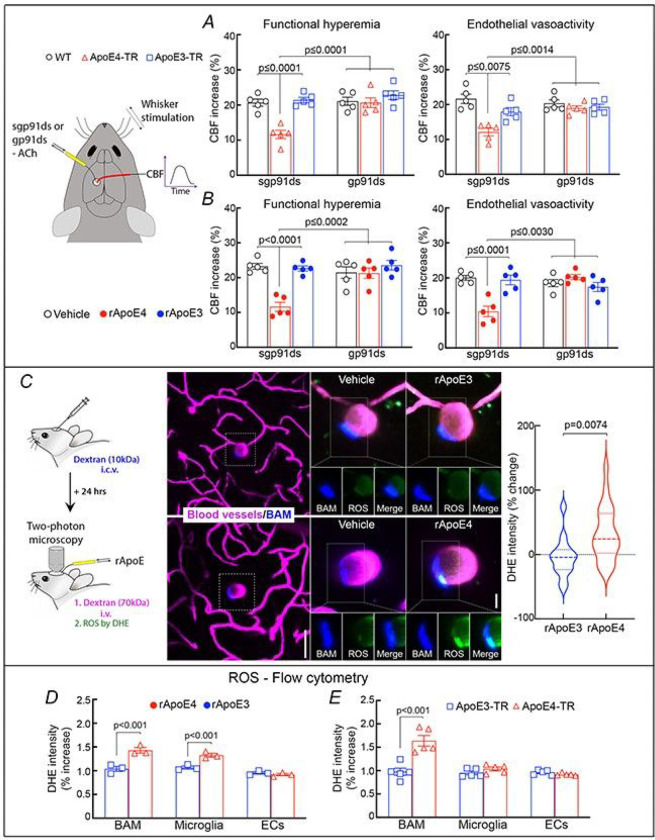
BAM mediate deleterious cerebrovascular effects of ApoE4 through NADPH oxidase-derived ROS. ***A.*** Neocortical superfusion of the NADPH oxidase peptide inhibitor gp91ds, but not its scrambled control (sgp91ds), rescues functional hyperemia and endothelial vasoactivity in ApoE4-TR mice. The peptide does affect CBF regulation in ApoE3-TR mice. ***B***. Pretreatment with gp91ds, but not sgp91ds, prevents the attenuation of functional hyperemia and endothelial vasoactivity induced by neocortical superfusion of rApoE4 in WT mice (10μg/ml). CBF responses are not attenuated by rApoE3 and the peptides have no effect. N=5/mice group. ***C***. *In vivo* ROS measurement by 2-photon microscopy. Left panel: WT mice received intracerebroventricular (i.c.v.) injection of dextran (10 kDa) to label border-associated macrophages (BAM, blue) and, 24 hrs later, intravenous infusion of the ROS marker dihydroethidium (DHE, green). Then, WT mice were equipped with an open cranial window and blood vessels were labeled with i.v. dextran (70 kDa, magenta). Middle panel: representative images illustrating that superfusion of rApoE3 (top) does not increase ROS (green), but superfusion with rApoE4 (bottom) increases the ROS signal. Right panel: quantification of ROS production in BAM; N=5 mice/group; 3–6 cells/mouse. ***D***. rApoE4 (10μg/ml), but not rApoE3, increases ROS production in BAM and microglia, but not in endothelial cells (ECs) from WT mice. ROS were measured *ex vivo* by flow cytometry. **E**. ROS production is higher in BAM of ApoE4-TR mice than in ApoE3-TR mice but not in microglia and ECs. Scale bars in **C**: 50 μm in left upper and lower panels and 10 μm in the enlarged images in the right panels; One-way ANOVA and Tukey’s test; data presented as mean±SEM.

The CBF dysfunction in ApoE4-TR mice is attenuated by a ROS scavenger ^33^. To provide insight into the enzymatic source of the radicals we examined the effect of a highly selective peptide inhibitor of NADPH oxidase (gp91ds-tat) ^50^, a major source of cerebrovascular oxidative stress ^51–53^. Pre-treatment of the cortex with gp91ds-tat did not influence baseline CBF responses but prevented the attenuation in functional hyperemia and endothelium-dependent vasoactivity in ApoE4-TR mice, while the scrambled control peptide had no effect (Fig. 2A). Similarly, gp91ds-tat counteracted the neurovascular dysfunction induced by neocortical application of rApoE4 in WT mice (Fig. 2B). CBF responses to adenosine were not affected in ApoE4-TR mice or with rApoE4 application in WT mice (Extended Data Fig. 1A). No changes in CBF responses were observed in ApoE3-TR mice or with rApoE3 application in WT mice (Fig. 2A,B; Extended Data Fig. 1A). These findings implicate NADPH oxidase-derived ROS in the mechanisms of the neurovascular dysfunction of ApoE4.

Next, we sought to define the cellular sources of the ROS. BAM have emerged as a leading cause of vascular oxidative stress via NADPH oxidase derived ROS ^40, 42, 43^. To investigate if these cells were the source of ROS, we first examined if rApoE4 induces ROS production in BAM. To this end, WT mice were injected intracerebroventricularly (icv) with cyanine-5 (Cy5)-dextran, which is phagocytosed by BAM rendering them fluorescent (Fig. 2C) ^40, 42, 44^. The following day, mice were anesthetized, equipped with a cranial window, injected i.v. with the ROS marker dihydroethidine (DHE) and with FITC-dextran to label cerebral microvessels ^42, 44^, and imaged with a 2-photon microscope ^43, 45^. Vehicle, rApoE3 or rApoE4 was applied to the exposed neocortex and ROS-dependent fluorescence was monitored in Cy-5-positive macrophages surrounding blood vessels at a depth of 150 μm from the pial surface. rApoE4 increased the ROS signal in BAM, but rApoE3 failed to do so (Fig. 2C). Furthermore, in WT mice rApoE4, but not rApoE3, increased ROS production, assessed by flow cytometry, in BAM (CD36^+^, CD45^high^) and microglia (CD45^Int^, CD11b^+^), but not in endothelial cells (Fig. 2D). In ApoE4-TR mice, the ROS signal was increased in ApoE4 BAM but not in ApoE4 microglia or endothelial cells (Fig. 2E). ROS were not elevated in ApoE3-TR mice (Fig. 2E). Of note, ROS increases in microglia were not observed in ApoE4-TR mice (Fig. 2E). These findings, collectively, demonstrate that ApoE4 triggers ROS production in BAM and that BAM are a major source of vascular oxidative stress in ApoE4-TR mice.

### 3. BAM are required for neurovascular dysfunction induced by ApoE4

**Figure 3:**
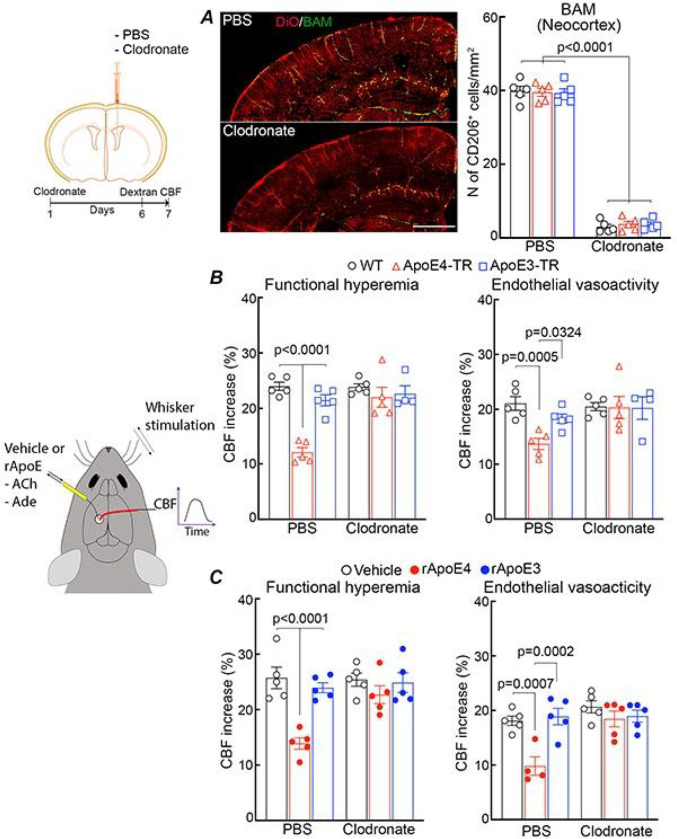
BAM depletion prevents the neurovascular dysfunction induced by ApoE4. ***A*.** BAM depletion by clodronate in WT mice. Mice were injected i.c.v. with PBS liposomes (vehicle) or clodronate and depletion was assessed 7 days later. BAM (green) surround blood vessels labeled by DiO (red; top panel). BAM numbers do not differ in WT, ApoE3-TR, and ApoE4-TR mice injected with vehicle. Clodronate depleted BAM equally in all groups (quantification in right panel). ***B***. BAM depletion prevents the attenuation in functional hyperemia and endothelial vasoactivity in ApoE4-TR mice compared to WT and ApoE3-TR mice. ***C***. BAM depletion counteracts the deleterious vascular effects of rApoE4 in WT mice. One-way ANOVA and Tukey’s test; N=5/group; data presented as mean±SEM.

Having established that the deleterious neurovascular effects of ApoE4 are mediated by NADPH oxidase-derived ROS and that ApoE4 induces ROS production in brain macrophages, we investigated if ApoE4 acts on BAM to mediate the neurovascular dysfunction. To this end, we depleted BAM using liposome-encapsulated clodronate (CLO) ^40, 42, 43^. CLO or liposome controls were injected icv in WT, ApoE3-TR and ApoE4-TR mice and, 5–7 days later mice were equipped for monitoring of CBF by laser-Doppler flowmetry. CLO depleted BAM by 80–90% in WT, ApoE3-TR and ApoE4-TR mice (Fig. 3A) without affecting microglia (Extended Data Fig. 1B), as shown before ^40, 42, 43^. In WT and ApoE3-TR mice, CLO did not alter functional hyperemia, endothelial vasoactivity to acetylcholine or CBF responses to the smooth muscle relaxant adenosine (Fig. 3B; Extended Data Fig. 1C). Remarkably, CLO completely reversed the attenuation of functional hyperemia and endothelium-dependent response in ApoE4-TR mice (Fig. 3B). Since application of rApoE4 to the exposed neocortex reaches leptomeningeal and perivascular spaces and increases ROS in BAM (Fig. 2C), we asked if CLO would also ameliorate the neurovascular dysfunction induced by neocortical application of rApoE4. CLO treatment did not affect neurovascular responses when vehicle or rApoE3 was applied to the neocortex, but completely prevented the attenuation in functional hyperemia and endothelial vasoactive function induced by rApoE4 application (Fig. 3C). CBF responses to adenosine were not affected (Extended Data Fig. 1C). These observations indicate that BAM are required for the neurovascular dysfunction induced by ApoE4.

### 4. BAM are the source of the ApoE mediating the neurovascular dysfunction

**Figure 4:**
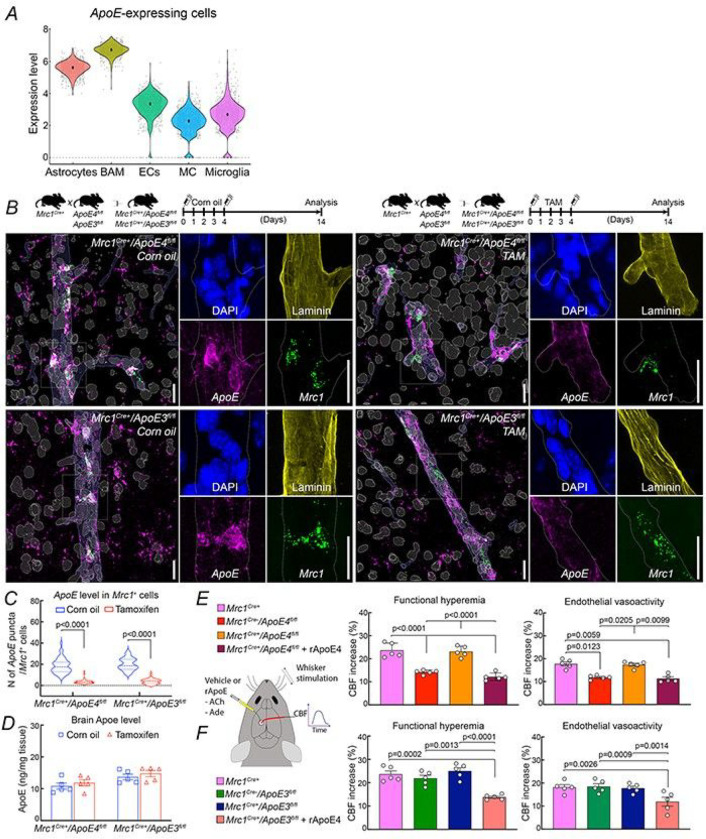
Deletion of ApoE4 selectively in BAM restores neurovascular function. ***A*.** BAM express *ApoE* at levels comparable to those of astrocytes but higher than microglia, endothelial cells, and cells of the vascular wall (MC, mural cells). ***B***. Dual RNAScope *in situ* hybridization with mRNA probes for *Mrc1* (green) and *ApoE* (magenta), combined with DAPI nuclear staining (blue) and the basement membrane marker laminin (yellow). Representative images illustrating abundant expression of *ApoE* in *Mrc1*^+^ cells in *Mrc1*^*Cre+*^*/ApoE4*^*fl/fl*^ and *Mrc1*^*Cre+*^*/ApoE3*^*fl/fl*^ mice treated with vehicle (corn oil). However, in *Mrc1*^*Cre+*^*/ApoE4*^*fl/fl*^ and *Mrc1*^*Cre+*^*/ApoE3*^*fl/fl*^ mice treated with tamoxifen (TAM) ApoE levels in *Mrc1*^+^ cells are markedly reduced. ***C***. *ApoE* puncta quantification in *Mrc1*^+^ cells; N=4–5 mice/group; 1–2-sections/mice; 5–12 cells/section. In ***B***, larger images on the left were reconstructed using Imaris software and smaller images on the right cropped from confocal photographs (see Extended Data Fig. 2*B-E*). Scale bars = 20 μm. ***D***. Brain ApoE levels, quantified by MSD, are comparable in vehicle and TAM-treated *Mrc1*^*Cre+*^*/ApoE4*^*fl/fl*^ and *Mrc1*^*Cre+*^*/ApoE3*^*fl/fl*^ mice (N=5/group). ***E-F***. BAM-specific deletion of *ApoE* restores functional hyperemia and endothelial vasoactivity in TAM-treated *Mrc1*^*Cre+*^*/ApoE4*^*fl/fl*^ mice (N=5/group) (***E***), but does not alter CBF responses in TAM-treated *Mrc1*^*Cre+*^*/ApoE3*^*fl/fl*^ mice (N=5/group) (***F***). rApoE4 markedly attenuates functional hyperemia and endothelial vasoactivity in TAM-treated *Mrc1*^*Cre+*^*/ApoE4*^*fl/fl*^ and *Mrc1*^*Cre+*^*/ApoE3*^*fl/fl*^ mice (N=5/group), attesting to the integrity of ApoE4 signaling pathways leading to neurovascular dysfunction despite BAM ApoE deletion. Data in ***C-F*** were analyzed using two-way ANOVA with Tukey’s test and are presented as mean±SEM.

Next, we asked if BAM are the source of the ApoE4 mediating the neurovascular dysfunction. In addition to astrocytes and disease-associated microglia ^47^, macrophages also express and secrete ApoE ^54^. To document that ApoE is also expressed in BAM we mined publicly available single-cell RNAseq databases of mouse brain for *ApoE* transcripts in astrocytes, myeloid and vascular cells (see methods). We found that *ApoE* is expressed in BAM at levels comparable to those of astrocytes, higher than microglia, endothelial cells, and cells of the vascular wall (Fig. 4A). ApoE expression in perivascular macrophages has also been observed in the human brain ^22^. Therefore, BAM could very well be the source of the ApoE4 mediating the neurovascular dysfunction.

To provide evidence that ApoE4 derived from BAM is responsible for the neurovascular dysfunction observed in ApoE4-TR mice we developed a mouse model to genetically target BAM. For tamoxifen-inducible Cre recombinase expression in BAM, we generated a new mouse line in which a *CreERT2* cassette was inserted into the Mrc1 locus (*Mrc1*^*CreERT2*^; here designated Mrc1^Cre^) (Extended Data Fig. 2A). To test the cellular selectivity of the Cre recombinase expression *Mrc1*^*Cre*^ mice were crossed with tdTomato mice (*R26*^*tdT*^: Jackson Laboratory Strain #: 007914). After tamoxifen treatment of *Mrc1*^*Cre+*^*/R26*^*tdT*^ crosses, recombinase activity was observed in over 80–90% of cells expressing the BAM marker CD206 (Extended Data Fig. 2A). No recombinase activity was seen in microglia or endothelial cells (Extended Data Fig. 2A).

To selectively knock-down ApoE4 in BAM we crossed *Mrc1*^*Cre*^ with *ApoE4*^*fl/fl*^ mice. The efficiency of the selectivity and downregulation for *ApoE* in BAM was verified with RNAscope *in situ* hybridization. In corn-oil (vehicle)-treated *Mrc1*^*Cre+*^*/ApoE4*^*fl/fl*^ or *Mrc1*^*Cre+*^*/ApoE3*^*fl/fl*^ mice, ApoE was observed both in BAM and in astrocytes, a major ApoE source (Fig. 4B,C; Extended Data Fig. 2B-C,F-G; Extended Data Fig. 3A,C,E-F). Then we went on to study the effect of *ApoE* deletion in BAM. Tamoxifen treatment of *Mrc1*^*Cre+*^*/ApoE4*^*fl/fl*^ or *Mrc1*^*Cre+*^*/ApoE3*^*fl/fl*^ mice deleted *ApoE* in BAM (Fig. 4B,C; Extended Data Fig. 2D-E), without altering *ApoE* in astrocytes (Extended Data Fig. 3B-F), or ApoE levels in brain, CSF or blood (Fig. 4D; Extended Data Fig. 3G,H). With tamoxifen treatment, neurovascular responses in *Mrc1*^*Cre+*^ mice were not different from those in WT mice, while in *Mrc1*^*Cre*−^*/ApoE4*^*fl/fl*^ mice functional hyperemia and endothelium-initiated CBF response were attenuated as much as in ApoE4-TR mice (Fig. 4E), as anticipated since *ApoE4*^*fl/fl*^ mice are equivalent to ApoE4-TR mice ^55^. Conversely, in tamoxifen treated *Mrc1*^*Cre+*^*/*^*/*^*ApoE4*^*fl/fl*^ mice neurovascular and endothelial responses were completely normal (Fig. 4F), but importantly application of rApoE4 to the neocortex reproduced in full the neurovascular dysfunction (Fig. 4E). This observation rules out the possibility that CBF responses were normalized because of deficits in ApoE receptors and/or downstream signaling pathways needed for ROS production. CBF responses to adenosine were not affected in all groups (Extended Data Fig. 1D). Similarly, rApoE4 attenuated neurovascular responses in *Mrc1*^*Cre+*^ mice, attesting to their susceptibility to the neurovascular effects of ApoE4 (Extended Data Fig. 4A). Of note, in the absence of tamoxifen, *Mrc1*^*Cre+*^*/ApoE4*^*fl/fl*^ exhibited attenuated CBF responses (Extended Data Fig. 4B), as did tamoxifen treated *Mrc1*^*Cre*−^*/ApoE4*^*fl/fl*^ mice (Fig. 4E) or ApoE4-TR mice (Fig. 3B), ruling out effects of tamoxifen. Confirming the link between ApoE4 and ROS production in BAM, the baseline ROS signal, assessed by DHE in cortical brain slices, was increased in BAM of *Mrc1*^*Cre+*^*/ApoE4*^*fl/fl*^ mice compared to *Mrc1*^*Cre+*^ mice and was reduced by treatment with tamoxifen (Extended Data Fig. 4C,D). As anticipated, neurovascular responses were not altered in tamoxifen treated *Mrc1*^*Cre+*^*/ApoE3*^*fl/fl*^ mice (Fig. 4F; Extended Data Fig. 1D). Therefore, downregulation of ApoE4 in BAM did not alter ApoE levels in the brain, but rescued the neurovascular dysfunction in full, consistent with BAM being the sole source of ApoE4 mediating the vascular effects.

### 5. BAM as source and target of the ApoE4 mediating neurovascular dysfunction

**Figure 5:**
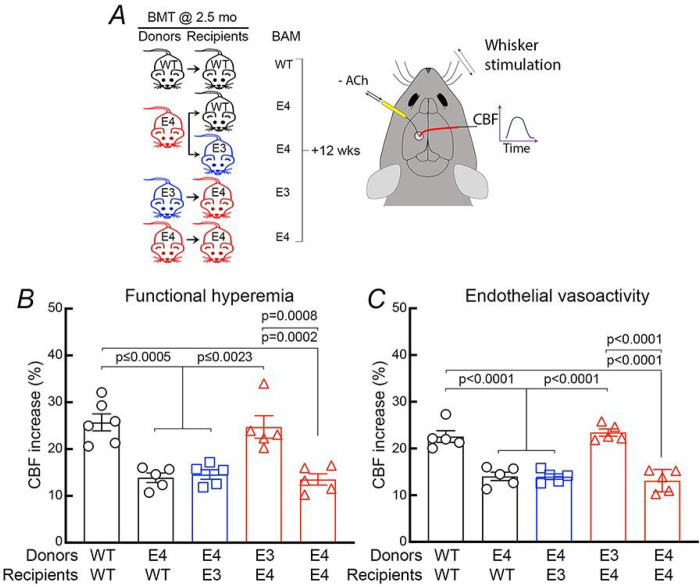
ApoE4 in BAM induces CBF dysfunction in ApoE3-TR mice, while ApoE3 in BAM reverses the dysfunction in ApoE4-TR mice. ***A*.** Mice received bone marrow transplantation (BMT) at 2.5 months of age and were studied 12 weeks later. ***B-C***. In WT mice transplanted with WT bone marrow (WT→WT) CBF responses are comparable to those in of naïve WT mice (see Fig. 1–3), whereas in ApoE4-TR transplanted with ApoE4 bone marrow (E4→E4) CBF responses are attenuated as in ApoE4-TR mice (see Fig. 2A). Remarkably, transplant of ApoE4 bone marrow into WT (E4→WT) or ApoE3-TR (E4→E3) mice, attenuates neurovascular responses as in ApoE4-TR mice, and, conversely, transplant of E3 bone marrow into ApoE4-TR mice (E3→E4) normalizes neurovascular function. Data in ***B-C*** were analyzed using one-way ANOVA with Tukey’s test and are presented as mean±SEM; N=5/group.

The results in *Mrc1*^*Cre+*^*/ApoE4*^*fl/fl*^ mice indicate that BAM are the source of ApoE4 mediating the vascular impairment. Furthermore, the observation that application of rApoE4 to pial and perivascular spaces in WT mice induces neurovascular dysfunction through BAM (Fig. 3C) suggests that BAM could also be target of ApoE4. If so, we hypothesized that repopulation of perivascular spaces of ApoE4-TR mice with ApoE3 macrophages would rescue the neurovascular dysfunction and, conversely, ApoE4 macrophages would induce the dysfunction in ApoE3-TR mice. To test this hypothesis, we used a bone marrow chimera-based approach. Bone marrow chimeras have been successfully used to study BAM ^40, 42, 43, 56, 57^. Brain irradiation depletes BAM and makes their niche accessible to bone marrow-derived macrophages, which repopulate the perivascular space and meninges, retain the ability to mediate vascular oxidative stress and neurovascular dysfunction ^40, 42, 43^ and are transcriptomically like BAM ^58^. Microglia are not replaced in this time frame ^40, 42, 44, 58, 59^. Using this strategy, we tested whether ApoE4 macrophages induce neurovascular dysfunction in an ApoE3-TR host, and, conversely, whether ApoE3 macrophages rescue the dysfunction in an ApoE4-TR host. Bone marrow from ApoE4-TR or ApoE3-TR mice was transplanted in ApoE3-TR (E4→E3TR) or ApoE4-TR mice (E3→E4TR), respectively. Experiments were performed 3 months after bone marrow transplantation, when the acute brain changes induced by irradiation have subsided and mice have an intact BBB, as well as neurovascular and cognitive function ^40, 42, 43, 60^. WT→WT or E4→E4TR bone marrow chimeras served as control to confirm lack of untoward effects of the transplantation procedure. As in previous studies ^40, 42^, neural, endothelial, or smooth muscle evoked CBF responses in WT→WT chimeras were not different from those of naïve WT mice (Fig. 5; Extended Data Fig. 1E), confirming that transplantation did not impair neurovascular regulation. Furthermore, in E4→E4TR chimeras CBF responses were attenuated as much as in ApoE4-TR mice (Fig. 5), indicating that bone marrow-derived macrophages repopulating perivascular and leptomeningeal spaces induce neurovascular dysfunction just like yolk sack-derived BAM. Remarkably, however, ApoE4-positive macrophages were able to induce neurovascular dysfunction in ApoE3-TR mice and, conversely, ApoE3-positive macrophages completely rescued the dysfunction in ApoE4-TR mice (Fig. 5). Therefore, ApoE3 in BAM normalize neurovascular function in ApoE4-positive host brains, whereas ApoE4 in BAM are sufficient to induce neurovascular dysfunction in ApoE3-positive host brains. These data provide evidence that the ApoE status of BAM is a key determinant of neurovascular function independently of the ApoE status of the host.

### 6. ApoE4 in BAM enhances white matter injury

**Figure 6:**
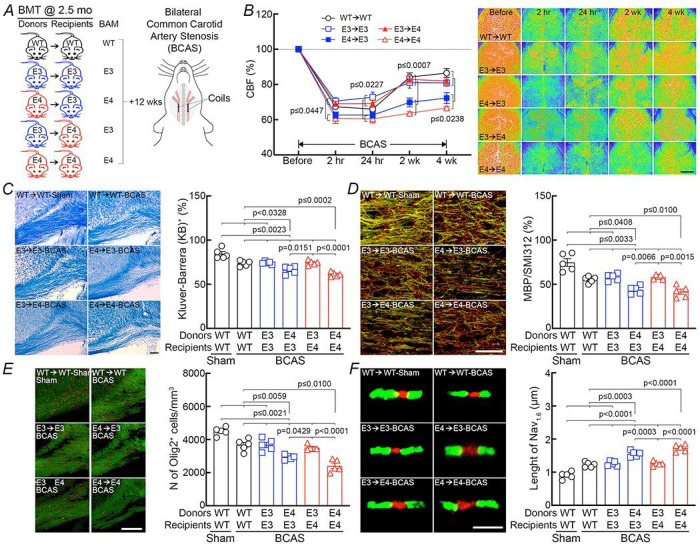
In a model of cerebral hypoperfusion ApoE4 in BAM worsens CBF reduction and white matter damage in ApoE3-TR mice, while ApoE3 in BAM ameliorates the phenotype. ***A***. Mice were transplanted as in Fig. 5A. Twelve weeks later, forebrain hypoperfusion was induced by bilateral common carotid artery stenosis (BCAS). ***B***. The reduction in neocortical CBF assessed by laser speckle flowmetry was worse in E4→E4 than in E3→E3 and WT→WT chimeras. However, E3→E4 BMT ameliorates the CBF reduction while E4→E3 BMT worsened it. N=5/group. Representative laser speckle images were shown from N=5/group. Scale bar = 2 mm. ***C***. Klüver-Barrera (KB) white matter stain of the corpus callosum in the same groups of mice in which CBF was assessed showing increased white matter damage in E4→E3 compared to E3→E3 chimeras, and reduced white matter damage in E3→E4 compared to E4^®^E4 chimeras. N=5 mice/group; scale bar = 100 μm. ***D***. Double-labeling immunofluorescence of myelin basic protein (MBP, green) and the pan-axonal neurofilament marker SMI312 (red) after BCAS illustrating a worsening of myelin integrity in E4→E3 chimeras, and improvement in E3→E4 chimeras. N=5 mice/group; scale bar = 100 μm. ***E***. Immuno fluorescence stain of MBP (green) and the oligodendrocyte marker Olig2 (red) illustrating a worse oligodendrocyte depletion in E4→E3 compared to E3→E3 chimeras, and an improvement in E3→E4 compared to E4→E4 chimeras. N=5 mice/group; scale bar = 100 μm. ***F***. Immunofluorescence stain of the nodal Nav1.6 channels (red) and the paranodal protein Caspr (green) showing increase nodal exposure in E4→E3 compared to E3→E3 chimeras, and an improvement in E3→E4 compared to E4→E4 chimeras. N=5/group; scale bar = 3 μm. In ***C-F***, representative images are shown on the left and related quantification on the right; representative images for each group are selected from 10 sections (2 sections per mouse) on which quantification was done. Data in *B* were analyzed with one-way ANOVA and Tukey’s test at each time point; data in *B-F*analyzed using two-way ANOVA with Tukey’s test; data are presented as mean±SEM.

**Figure 7:**
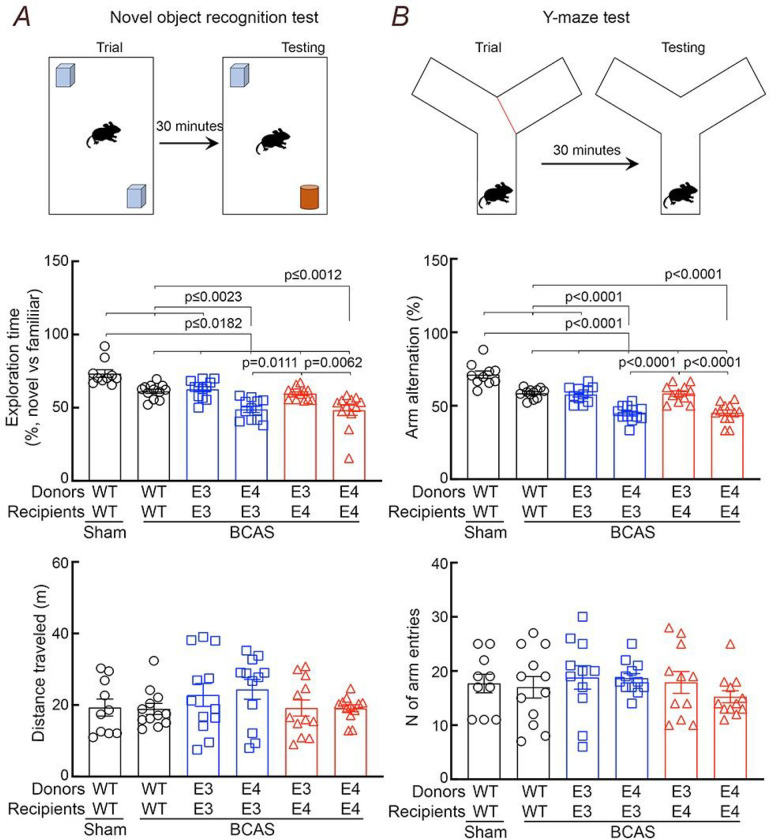
In a model of cerebral hypoperfusion ApoE4 in BAM worsens cognitive deficits in ApoE3-TR mice, while ApoE3 in BAM ameliorates the cognitive phenotype. In agreement with the CBF and WM damage data, E4→E3 chimeras exhibit worse cognitive deficits than E3→E3 chimeras at the novel object recognition (***A***) and Y-maze (***B***) tests, while E3→E4 chimeras exhibit cognitive improvement compared to E4→E4 chimeras. Indices of locomotor activity, recorded during the novel object recognition test (distance traveled) or the Y-maze test (number of arm entries), do not differ among groups. Data in ***A*** *and* ***B*** were analyzed with two-way ANOVA and Tukey’s test and are presented as mean±SEM. N=10–12/group.

Finally, we wondered if ApoE4 in BAM is also involved in the exacerbation of WM damage observed in ApoE4-TR mice ^33^. To this end, we induced chronic white matter damage to the corpus callosum by reduction in CBF using BCAS, a well-established model of oligemic white matter injury ^61^, in ApoE bone marrow chimeras. Stainless-steel coils, 0.18mm in diameter ^33, 62^, were placed around both common carotid arteries to reduce the vessel’s lumen and lower forebrain CBF in WT→WT, E3→E3TR, E4→E3TR, E3→E4TR, and E4→E4TR chimeras (Fig. 6A). In agreement with previous data in ApoE3-TR and ApoE4-TR mice ^33^ the CBF reduction, assessed by laser-speckle imaging, was comparable in WT→WT, E3→E3TR, but was more pronounced in E4→E4TR chimeras (Fig. 6B). However, in E4→E3TR the CBF reduction was comparable to that of E4→E4TR chimeras, and in E3→E4TR comparable to that of E3→E3TR chimeras (Fig. 6B). In all groups, BCAS induced white matter injury, as shown by the Kluver-Barrera stain, and by loss of myelin basic protein and oligodendrocytes (Fig. 6C–E). We also noticed loss of the integrity of the nodes of Ranvier, assessed by the spatial relationship between the paranodal contactin-associated protein Caspr and the nodal sodium channel Nav1.6 ^33^ (Fig. 6F). Cognitive testing by the novel object recognition and Y-maze tests also showed impairment (Fig. 7A,B), as reported previously ^33^. In agreement with the CBF data, E4→E3TR chimeras had worse white matter damage and cognitive deficits, which did not differ from those in E4→E4TR chimeras, while the outcome in E3→E4TR chimeras was comparable to that of E3→E3TR chimeras (Fig. 7A,B).

## Discussion

We investigated the role of BAM in the neurovascular dysfunction and increased susceptibility to white matter injury associated with the ApoE4 genotype. We demonstrated that ApoE4 alters critical homeostatic neurovascular regulatory mechanisms through NADPH oxidase-derived ROS, and that BAM are a pathogenic source of ROS in ApoE4-TR mice and in WT mice exposed to rApoE4. We also showed that BAM depletion normalizes neurovascular function in ApoE4-TR mice and prevents the dysfunction in rApoE4-treated WT mice. Selective deletion of ApoE4 in BAM using a novel *Mrc1*^*Cre+*^*/ApoE4*^*fl/fl*^ mouse did not alter ApoE levels in brain, CSF or blood, but completely rescued the neurovascular alterations, consistent with BAM being the sole source of ApoE targeting the microvessels. Using a bone marrow chimera-based strategy we found that ApoE4-positive BAM cause neurovascular dysfunction in ApoE3-TR mice, while ApoE3-positive BAM rescue in full the dysfunction in ApoE4-TR mice. Consistent with these vascular findings, in an oligemic model of chronic white matter injury, ApoE4-positive BAM worsened the flow reduction, white matter damage, and cognitive dysfunction in ApoE3-TR mice, while ApoE3-positive BAM fully reversed these effects in ApoE4-TR mice. These findings, collectively, unveil a critical role for BAM in the vascular effects of ApoE4 and identify these cells as both the source and the target of the ApoE4 mediating the dysfunction (Extended Data Fig. 5).

ApoE is produced by different cell types in brain, major sources including astrocytes and disease-associated microglia ^47^, and, as also shown here, BAM. However, our data demonstrate that cellular sources of ApoE4 other than BAM do not contribute to neurovascular dysfunction in ApoE4-TR mice. First, BAM depletion rescues vascular function in ApoE4-TR mice, in which other cell types producing ApoE4 are still present. Second, selective ApoE4 deletion in BAM does not reduce brain or blood levels of ApoE but rescues neurovascular function in full, indicating that ApoE4 from other sources does not play a role. Third, ApoE3-positive BAM are sufficient to normalize neurovascular function in an ApoE4 host, and, conversely, ApoE4-positive BAM are sufficient to induce neurovascular dysfunction in an ApoE3 host. Therefore, the data reveal a previously unappreciated perivascular compartmentation of the ApoE4 acting on the vasculature, which is probably related by the proximity of the ApoE4-ROS source to the vasculature, i.e., BAM abutting resistance arterioles regulating cerebral perfusion ^35^. Whether such compartmentation also applies to sources of ApoE4 involved in other aspects of ApoE4 pathobiology, for example exacerbation of amyloid, tau or synuclein pathology ^63–65^, remains to be established.

Previous studies have shown that ApoE4 expression in endothelial and smooth muscle cells or in the periphery can compromise neurovascular integrity ^66–68^. These elegant studies have provided further evidence of the powerful vascular effects of ApoE4 but could not determine the targets of ApoE4, the cellular mechanisms of the vascular dysfunction nor the pathogenic consequence for the subcortical white matter when brain perfusion was threatened. Our data establish that in ApoE4-TR mice BAM are the unique effectors of the neurovascular dysfunction and susceptibility to white matter damage, in a cell-autonomous manner. However, it remains unclear whether in pathological conditions other vascular or systemic sources of ApoE4 may also play a role.

The findings have significant implications for the understanding of the increased propensity to microvascular alterations observed in ApoE4-positive individuals, including white matter damage and the ARIA syndrome in the setting of anti-Aβ immunotherapy ^23–25, 69^. Our data raise the possibility that ApoE4 derived from BAM may contribute to these conditions and as such could be a target for therapeutic interventions. However, it is conceivable that in the presence of Alzheimer pathology or vascular risk factors, such as hypertension, ApoE4 originating from microglia or astrocytes, could also be involved. Studies addressing the role of ApoE4 in BAM and other cell types in pathological states may provide the opportunity to answer this question.

In conclusion, we demonstrated a new cell autonomous role of BAM in the neurovascular dysfunction of ApoE4 and provided evidence for a previously unrecognized perivascular compartmentation of the cellular source of the ApoE4 targeting the cerebral microvasculature and enhancing white matter injury. The findings expand our understanding of the role of BAM in neurovascular diseases leading to cognitive impairment, and provide a putative target to counteract the increased susceptibility to white matter injury and microvascular pathology associated with the ApoE4 genotype.

## Materials and Methods

### Mice

All procedures were approved by the Institutional Animal Care and Use Committee of Weill Cornell Medicine and performed according to the ARRIVE guidelines ^70^ and, as much as feasible, in a blinded fashion. Experiments were performed in homozygous ApoE3-TR and ApoE4-TR mice on a C57BL/6 genetic background ^71^. ApoE3-floxed (*ApoE3*^*fl/fl*^) and ApoE4-floxed (*ApoE4*^*fl/fl*^) homozygous mice on a C57BL/6 genetic background ^55^ were crossed with *Mrc1*^*CreERT2*^ mice (see “Generation of the Mrc1 targeting construct”). C57BL/6 mice were used as wild-type (WT) controls. We use male and female mice aged 3–6 months.

### Generation of the Mrc1 targeting construct

*Mrc1*^*CreERT2*^ mice (*Mrc1*^*Cre+*^) were generated by Cyagen. The *Mrc1* targeting construct (Extended Data Fig. 2A) was linearized by restriction digestion with NotI, followed by phenol/chloroform extraction and ethanol precipitation. The linearized vector was transfected into C57BL/6 ES cells according to Cyagen’s standard electroporation procedures. The transfected ES cells were subject to G418 selection (200 μg/mL) 24 hours post electroporation. Total 179 G418 resistant clones were picked and amplified in 96-well plates. Two copies of 96-well plates were made: one copy for storage at −80°C and the other copy for DNA isolation and subsequent PCR screening for homologous recombination. The PCR screening identified 18 potential targeted clones, from which 6 were expanded and further characterized by Southern blot analysis. Five of the six expanded clones were confirmed to be correctly targeted. The *Mrc1* gene (NCBI Reference Sequence: NM_008625.2) is located on mouse chromosome 2. Thirty exons have been identified, with the ATG start codon in exon 1 and the TAG stop codon in exon 30. In the targeting vector, the coding region of exon 1 plus part of intron 1 was replaced with the CreERT2-polyA cassette. Correct integration of the CreERT2-polyA cassette was confirmed by diagnostic PCR. Sequencing of the genomic region of interest after amplification by PCR was further used to verify correct integration. After germline transmission, correctly targeted founders (*Mrc1*^*Cre+*^ mice) were crossed with C57BL/6J mice to establish colonies for this study. To assess *Mrc1* Cre recombinase in *Mrc1*^*Cre+*^ mice, we crossed with tdTomato mice (*R26*^*tdT*^: Jackson Laboratory Strain #: 007914), activated Cre recombinase with tamoxifen (TAM; Sigma, T-5648) dissolved in corn oil (Sigma, C-8267), and assessed the labeling efficacy. In 8–10-week-old mice, Cre recombination was induced by injecting 5.mg TAM subcutaneously for 5 consecutive days. Then, CD206^+^ BAMs were counted and analyzed (Extended Data Fig. 2A). At least three sections from a minimum of three mice were used for each analysis. Quantification on three-dimensionally reconstructed CD206^+^ cell images was obtained using Imaris software.

### ApoE expression in brain myeloid and vascular cells

Mouse brain single cell mRNA data were extracted from the GSE174574 dataset deposited in the Gene Expression Omnibus repository (https://www.ncbi.nlm.nih.gov/geo/). The data used was derived from three male C57BL/6 mice (6–8 weels of age) that underwent sham surgery for middle cerebral artery occlusion ^72^. We chose this dataset because it contains brain myeloid, endothelial, mural cells, and astrocytes. After quality filtering that removed cells with <500 and >20,000 transcripts and more than 20% reads from mitochondrial genes, the count matrices were processed with Seurat (Vers. 4.1.0) ^73^ in the R statistical environment. Log normalization, variable gene detection, scaling, and principal component analysis was performed with default settings. The R package Harmony ^74^ was used to correct the matrix for batch effects. We used the Louvain algorithm as implemented in Seurat *FindClusters* to perform graph-based clustering on the neighbor graph that was constructed with the *FindNeighbors* function call on Harmony-derived embeddings. We performed unsupervised cell type annotation using the SingleR package ^75^ with ImmGen ^76^, BrainImmuneAtlas ^77^, and Tabula Muris ^78^ as reference datasets. Data were visualized in R using ggplot2 (V3.3.6).

### Intracerebroventricular injection of clodronate or dextran

Liposomes containing clodronate or PBS were administered intracerebroventricularly as previously described ^40, 42, 43^. Isoflurane-anesthetized mice were placed in a stereotaxic frame. Ten microliters of clodronate liposomes (7 mg/mL) or PBS liposomes (vehicle) were injected into the cerebral ventricles with a glass micropipette (rate <0.5 μl/min) through a burr hole drilled on the right parietal bone. Mice were used in the experiments 5 to 7 days later, when BAM depletion is well developed and stable ^40, 42, 43^. In some experiments, BAM were identified by their ability to phagocytize dextran ^40, 42^. For dextran injections, 10 μl of Alexa Fluorâ 680 dextran (10,000 MW, anionic, fixable, ThermoFisher Scientific, D34680; 2.5 mg/ml) in PBS or PBS alone were slowly injected into the cerebral ventricles with a glass micropipette through a burr hole drilled on the right parietal bone ^42^. BAM labeling was examined 24 hrs later.

### Labeling cortical blood vessels with DiO

Cortical blood vessels were labeled with the lipophilic dye DiO [DiOC18(3) (3,3’-Dioctadecyloxacarbocyanine Perchlorate)], as described ^42, 44^. Briefly, mice were anesthetized (5% isoflurane) and transcardially perfused with PBS (2 ml) followed by DiO (1:50, V-22886, Molecular Probes; 5ml/mouse) and then by 4% paraformaldehyde (PFA). Brains were harvested and post-fixed in 4% PFA overnight, then cut (thickness 150 μm) using a vibratome and examined under the confocal microscope (Leica SP8).

### Lipidation of recombinant ApoE3 and ApoE4

Recombinant (r) ApoE3 (Cat# A128, Leinco Technologies) and rApoE4 (Cat# A129, Leinco Technologies) were purchased and lipidated as previously described ^79^. Briefly, lyophilized rApoE3/4 was solubilized in DPBS buffer with 1mM DTT and 1mM EDTA at a final concentration of 25 μM. Separately, POPC (Avanti Polar Lipids) and cholesterol (Avanti Polar Lipids) dissolved in chloroform were combined in a glass vial for a final molar ratio of ApoE:POPC:Cholesterol at 1:50:10. The POPC/cholesterol mixture was dried under nitrogen until chloroform was completely evaporated. Then, DPBS was added to the dried POPC/cholesterol mixture and allowed to hydrate for 30 minutes. The mixture was added with sodium cholate (Sigma) at a 4:1 ratio (g/g) sodium cholate:POPC and incubated for 1 hour. Next, rApoE3/4 were added to sodium cholate:POPC:cholesterol mixture and incubated for 1hr at room temperature. The mixture was dialyzed in PBS at 4°C for 48 hrs using a 10,000 MWCO Slide-A-Lyzer Dialysis Cassette (Thermo Scientific) with three buffer changes. Samples were purified using a Superose 6 10/300 Increase GL column (Cytiva) with a flow rate of 0.5mL/min in PBS buffer. Samples were concentrated using an Amicon Ultra-15 10,000 MWCO concentrator (Sigma) at 4,000g for 20min at 4°C.

### Monitoring of cerebral blood flow.

CBF was monitored using laser-Doppler flowmetry or laser-speckle flowmetry as previously described ^33, 40, 42, 45^ and briefly summarized below.

#### CBF response to neural activity and to endothelium-dependent and independent agonists:

As described in detail previously ^42, 45, 80^, anesthesia was induced with isoflurane (1–2%) and maintained with urethane (750 mg/kg; i.p.) and a-chloralose (50 mg/kg; i.p.). A femoral artery was cannulated for recording of arterial pressure and collection of blood samples. A 2×2 mm opening was drilled in the parietal bone overlying the somatosensory cortex, the dura was removed, and the site was superfused with a modified Ringer solution (37°C; pH 7.3–7.4) ^40, 45^. Relative CBF was continuously monitored at the site of superfusion with a laser-Doppler flowmeter (Perimed). Arterial blood pressure, blood gases and rectal temperature were monitored and controlled. CBF recordings were started after arterial pressure (MAP, 78–85 mmHg) and blood gases (pO_2_, 120–140 mmHg; pO_2_, 33–40 mmHg; pH, 7.3–7.4) were in a steady state^45, 80^. For functional hyperemia, the whiskers were mechanically stimulated for 30 sec and the associated increase in CBF was recorded. To test endothelium-dependent responses ACh (100 μM; Sigma) was superfused on the cranial window, and the resulting change in CBF recorded. The CBF response to superfusion with adenosine (400 μM; Sigma) was also tested. To test the effect of topical application of recombinant (r) ApoE3 or rApoE4 on functional hyperemia, ACh, and adenosine, the cranial window was superfused with a Ringer’s solution containing rApoE4 (Cat# A219, Leinco Technologies) or rApoE3 (Cat# A218, Leinco Technologies) and lipidated rApoE4 or rApoE3 (see lipidation section above). Lipidated or non-lipidated rApoE was reconstituted in PBS and then diluted in normal Ringer’s solution. The CBF response to whisker stimulation, ACh, or adenosine was tested 40 min after rApoE superfusion. In some experiments, the CBF response to whisker stimulation, ACh, or adenosine was tested before and 40 min after superfusion of the cranial window with the ApoE receptors inhibitor receptor-associated protein (RAP, 200 nM; Molecular Innovations), gp91ds-tat (gp91ds, 1 μM; Cat# AS-63818, AnaSpec), or scrambled gp91ds-tat (sgp91ds, 1 μM; Cat# AS-63821, AnaSpec).

#### Chronic CBF recordings after BCAS:

CBF was monitored with laser-speckle imaging (Omegazone; Omegawave). Mice were anesthetized with 1–2% isoflurane, and the scalp was removed to expose the skull. The following day, mice were re-anesthetized and the exposed skull was illuminated by with laser light (780 nm). The scattered light was filtered and detected by a CCD camera positioned over the skull. The raw speckle images were used to compute speckle contrast, which in the mouse neocortex reflects the velocity of moving red blood cells up to a depth of ≈700μm. Color-coded blood flow images were obtained in high-resolution mode (639 × 480 pixels; 1 image/sec) and the sample frequency was 60 Hz. One CBF image was generated by averaging numbers obtained from 20 consecutive raw speckle images. The recordings were initiated after the CBF was stable, and five recordings of blood flow image were averaged. The CBF reduction induced by BCAS was calculated as a percentage of the pre-stenosis CBF value. CBF changes were recorded 2hrs, 24hrs, 2 weeks, and 4 weeks after BCAS.

### ROS measurement

ROS production was assessed with 2-photon microscopy *in vivo*, flow cytometry or in brain slices using dihydroethidine (DHE) as a marker ^40, 42, 44^.

#### 2-photon microscopy:

BAM were labeled with an intracerebroventricular injection of 10 μL of Alexa Fluor 647 dextran (10 000 MW, anionic, fixable, ThermoFisher Scientific, Cat# D22914; 2.5 mg/mL) in PBS as described above ^40, 42, 44^. The next day, mice were briefly anesthetized with isoflurane (1.5–2%) and injected i.v. with dihydroethidium (10 mg/kg; ThermoFisher; Cat # D11347). One hour later, mice were re-anesthetized with isoflurane (1.5–2%) and equipped with a cranial window superfused with Ringer as described in the CBF experiments. Mice were injected retro-orbitally with fluorescein dextran-conjugated dye (2.5% w/v FITC 70 kDa) diluted in sterile saline (50 μl) to visualize the vasculature ^43, 45^ and imaged under a two-photon microscope (Fluoview FVMPE, Olympus) with a solid-state laser (InSight DS+; Spectra physics) set to an 820 nm wavelength. Image stacks were acquired through Fluoview software (FV31S-SW, v.2.3.1.163, Olympus). A map of the vasculature was taken through an x5 objective (MPlan N 5 × 0.1 NA, Olympus) to identify vessels branching from pial arteries at the cortical surface that feed the barrel area. Once the blood vessels to be imaged were identified, we switched to a 25x objective (XLPlan N 25 × 1.05 NA, Olympus) to identify BAM along the penetrating blood vessels. Then, z-stack images (509 × 509 μm^2^; 800 × 800 pixels) were acquired at <150 μm depth from the surface of the brain. The superfusion solution was switched from normal Ringer to Ringer containing rApoE3 (10 μg/ml; Cat# A218, Leinco Technologies) or rApoE4 (10 μg/ml; Cat# A219, Leinco Technologies). This concentration as chosen to assure sufficient penetration of ApoE into the neocortex. Forty minutes later, second images were acquired. Images were analyzed using ImageJ software. DHE fluorescence intensity in dextran-positive cells was compared before and after rApoE superfusion.

#### Flow cytometry:

Isolation of brain cells was performed as described ^43, 81^. Mice were anesthetized with pentobarbital (100 mg/kg, i.p.) and transcardially perfused with heparinized PBS. Brain cell isolation was performed by enzymatic digestion with Liberase DH (Roche Diagnostics) and Dispase (Worthington). Brain hemispheres were separated from the cerebellum and olfactory bulb and gently triturated in HEPES-HBSS buffer containing the following: 138mM NaCl, 5mM KCl, 0.4mM Na_2_HPO_4_, 0.4mM KH_2_PO_4_, 5mM d-glucose, and 10mM HEPES using a Gentle MACS dissociator (Miltenyi Biotec) following the manufacturer’s instructions. The suspension was digested with 125 μg/ml Liberase, 0.8U/ml dispase, and 50 U/ml DNase I at 37°C for 45 min in an orbital shaker at 100 rpm. Brain cells isolated were washed and subjected to 30% Percoll (GE Healthcare) density gradient centrifugation at 500g for 15 min, and washed with 20.mL 2% FBS in PBS and centrifuged at 500*g* for 7 min. Following isolation of brain cells, cells were incubated with dihydroethidium (DHE, 2.5mM) in stimulation buffer (RPMI-1640, 10% (v/v) heat inactivated FBS, 100 units/mL penicillin, 100 μg/mL streptomycin) for 30 minutes at 37° and 5% CO_2_. Some cells were pooled and separated for stimulation experiments, and were incubated with PBS, human rApoE3 or rApoE4 (10μg/mL) for 30 minutes prior to addition of DHE (as above). Cells were washed with flow cytometry buffer (1X PBS, 2% FBS, 0.05% NaN_3_) and centrifuged at 500*g* for 7 min. For surface marker analysis, 1 × 10^6^ cells approximately were resuspended in 50 μL of flow cytometry buffer. Cells were blocked with anti-CD16/CD32 for 10 min at 4 °C and then stained with the appropriate antibodies for 15 minutes at 4 °C. Cells were washed with flow cytometry buffer, resuspended in 200 μL of flow cytometry buffer and acquired with NovoSampler Q (NovoCyte Quanteon), and absolute cell numbers and frequencies were recorded. Samples were analyzed with FlowJo (Vers.10, Tree Star). Appropriate isotype controls, “fluorescence minus one” staining, and staining of negative populations were used to establish sorting parameters. Endothelial cells were identified as CD45^−^Ly6C^+^, microglia were identified as CD45^int^CD11b^+ 43, 81^, and BAM were identified as CD45^hi^CD11b^+^CD36^+ 43, 81^. Antibodies used were CD16/CD32 (clone 93, rat IgG2b, PRID: AΒ_312800; Biolegend), CD45 (label: BV510, clone 30F-11, rat IgG2b κ, PRID: AΒ_2561392; Biolegend), Ly6C (label: FITC, clone HK1.4, rat IgG2c κ, PRID: AΒ_1186134; Biolegend), CD11b (label: PECy7, clone M1/70, rat IgG2b κ, PRID: AΒ_830641; Biolegend), and CD36 (Label: FITC/PE, clone MF3, rat IgG2a, Cat # MA5-528168/MA5-16832; Invitrogen).

#### Brain slices:

Following euthanasia, the whole brain was quickly removed and submerged into ice-cold (4°C) oxygenated (95% O_2_–5% CO_2_) sucrose-aCSF (s-aCSF) and sectioned in 200 μm thickness using a vibratome (Leica VT1000). The s-aCSF was composed of the following (in mM): 248 sucrose, 5 KCl, 26 NaHCO_3_, 5 MgSO_4_, 1 NaH_2_PO_4_, 0.5 CaCl_2_, and 10 glucose, pH=7.35. The lactic acid (l)-aCSF was composed of the following (in mM): 124 NaCl, 5 KCl, 26 NaHCO_3_, 2 MgSO_4_, 2 CaCl_2_, 1 NaH_2_PO_4_, 10 glucose and lactic acid 4.5, pH=7.35. Brain slices were started to cut from the bregma. Once the slice free-floated, it was transferred to an incubation chamber filled with l-aCSF oxygenated with 95% O_2_–5% CO_2_. Brain slices were then allowed at least 60 minutes to recover from slicing followed by loading for 45 minutes with the ROS indicator DHE (2 μM) in oxygenated l-aCSF buffer. DHE fluorescent intensity was measured using a Bromide HE filter in Cy-5 labeled BAM along the penetrating vessels in neocortices. Using IPLab software (Scanalytics, Fairfax, VA), time-resolved fluorescent intensity was acquired at 5-min intervals with an exposure time of 100-msec for 20-min using a Nikon Diaphot 300 inverted microscope equipped with a CCD digital camera (Princeton Instruments, Trenton, NJ). BAM ROS (DHE intensity) production was assessed before and after ApoE superfusion and the change was expressed as relative fluorescence units. Data were acquired from N=3–4 mice per group, 1–2 brain slices/mouse, and 3–7 cells/slice.

#### *in situ* hybridization

Tissue was prepared for the RNAScope Multiplex Fluorescent Assay according to the manufacturer’s instructions (Cat # 323100, ACD, Hayward, CA). The tissue was sectioned (20 μm) in a cryostat and mounted on charged slides. Slides were pretreated with target retrieval buffer (Cat # 322000) boiled for 5 min and treated with Protease III at 40 °C for 30 min (Cat # 322381), followed by incubation with mRNA probes for 2 h at 40 °C. The probes used were *Mrc1* (Cat # 437511-C1), *ApoE* (Cat # 433091-C3), and *GFAP* (Cat # 486191-C2). A three-plex positive control probe (Cat # 320881) and three-plex negative control (Cat # 320891) were used to ensure the RNAScope positivity. After incubating with the probes, sections were counterstained with the blood vessel marker laminin (anti-rabbit; Cat # ab11575, abcam) followed by incubation with the nuclei marker DAPI (Cat # D1306, Thermo Fisher) and Cy3-conjugated secondary antibody. Then, slides were coverslipped in Prolong Gold Antifade Mountant (Thermo Fisher, Cat # P36930) and, after drying, images were acquired with Leica confocal microscope and analyzed with NIH ImageJ. Some sections were further processed and visualized using Imaris software.

### ApoE measurement

ApoE was measured with an electrochemiluminescence-based multi-array method through the Quickplex SQ 120 system (Meso Scale Diagnostics LLC). After the CBF experiment, CSF samples were collected via a cisterna magna puncture. Blood samples were collected in a tube containing sodium citrate (3.8%) through the femoral artery catheter. Sodium citrate was added (1:9 ratio) to the blood samples. Plasma was collected following centrifugation at 2,000 × g for 15 minutes. Brain tissues were harvested following transcardiac perfusion of saline. They were homogenized with a sonicator in cold RAB buffer at 10 μl/mg and centrifuged at 50,000 × g for 20 minutes at 4°C. The supernatant was saved, and the pellet was homogenized using a sonicator in cold RIPA buffer at 10 μl/mg and centrifuged at 50,000 × g for 20 minutes at 4°C. The supernatant was then collected. According to the manufacturer’s protocol, human ApoE levels were quantified using the MSD R-PLEX human ApoE assay (Cat# K1512IR-2).

### Bone marrow transplantation

Procedures for BM transplantation have been previously described ^40, 42, 44^ and are only summarized. Whole-body irradiation was performed in 10-weeks-old mice (Nordion Gammacell 40 Exactor). Eighteen hours later, mice were transplanted with BM cells (2×10^6^, i.v.) isolated from ApoE3-TR, ApoE4-TR, and WT controls. Mice were housed in cages with sulfamethoxazole (0.12%; w/v) and trimethoprim (0.024%) added to drinking water for the first two weeks. Reconstitution of BM cells was verified 12 weeks after irradiation by testing the positive human ApoE3 and ApoE4 genomic DNA percentage in isolated blood leukocytes. Reference primers sequences were as follows: m_ICAM1_prom.3, 5′-GGACTCACCTGCTGGTCTCT-3′ and m_ICAM1_prom.4, 5′-GAACGAGGGCTTCGGTATTT-3′; target primers sequences were as follows: CD36_1, 5’- -3’ and CD36_2, 5’- -3’, m_Cybb_gt_1, 5’-CTGCTCACCAGCCTCTCTCTA-3’ and m_Cybb_gt_2, 5’-CTGGAACCCCTGAGAAAGGAG-3’ (Invitrogen). qRT-PCR was conducted with 20 ng of DNA, in duplicate 15 μl reactions using the Maxima SYBR Green/ROX qPCR Master Mix (2×) (Thermo Scientific). Chimerism was >95% for BM chimeras of ApoE3-TR, ApoE4-TR, and WT controls. A PCR cycling protocol consisting of 15 s at 95°C and 1 min at 60°C for 45 cycles was used for quantification. Human ApoE3 and ApoE4 relative expression levels were calculated by the 2 (−ΔΔ CT) method. To study BAM number and distribution after BM transplant in mice, BM from mice expressing GFP (GFP BM) was transplanted into ApoE3/4-TR mice or WT littermates at 10 weeks of age and the brain distribution of GFP expressing cells was examined at 22 weeks of age.

### Bilateral common carotid artery stenosis

As described previously ^33^, mice were anesthetized with isoflurane (1–2%) in a mixture of oxygen-nitrogen with rectal temperature maintained at 37°C. Both common carotid arteries were dissected thorough a midline incision and microcoils (internal diameter: 0.18 mm; Sawane Spring, Japan) were placed around the arteries ^33^. Sham-treated mice underwent the same surgical procedure with no placement of microcoils.

### Evaluation of white matter injury

Four weeks after BCAS, anesthetized mice were perfused transcardially ^33, 45, 82^. Brains were removed, postfixed overnight, and sectioned with cryostat (thickness of 12 μm) or vibratome (40 μm). For immunohistochemical evaluation of BCAS-induced WM injury the following antibodies were used with the appropriate secondary antibodies: anti-myelin basic protein (MBP) (rat; 1:500; Millipore Sigma), the neurofilament anti-SMI312 (mouse;1:500, Covance), anti-contactin-associated protein (Caspr) (mouse; 1:300, Millipore Sigma) ^33^, anti-voltage-gated sodium channel (Na_v_) 1.6 (Na_v_ 1.6) (rabbit; 1:200, Alomone) ^33^, or anti-oligodendrocyte transcription factor 2 (Olig2) (rabbit; 1:200; Millipore Sigma). Images were obtained with a confocal laser-scanning microscope (Leica SP5) and analyzed using ImageJ. The Klüver-Barrera stain was performed using the Luxol Fast Blue Stain Kit (ScyTek Laboratory Inc.). Brains were harvested after transcardiac perfusion with PBS and 4% PFA, sectioned with a vibratome (thickness 40 μm), and the positive (blue stained) area in the CC was quantified by ImageJ.

### Cognitive testing

Methods for cognitive testing have been described previously ^33, 40, 45, 82^. We elected to use the Y-maze and novel object recognition because these tests: (a) are sensitive to the cognitive effects of BCAS ^33, 83–85^, (b) rely on white matter tracts connecting the hippocampus to the cortex and other brain regions ^86^ and, as such, are appropriate in a model of white matter damage, (c) rely on the spontaneous behavior of mice and (c) do not required aversive environments or starving of the mice ^87, 88^.

#### Y-maze spontaneous alternation behavior:

Mice were placed into one of the arms of the maze (start arm) and allowed to explore only two of the three arms for 5 min (training trial). The closed arm was opened in the test trial, serving as the novel arm. After a 30-min interval between trials, the mice were returned to the same start arm and were allowed to explore all three arms for 5 min (test trial). Sessions were video recorded and analyzed using AnyMaze (San Diego Instruments) in a double-blinded fashion. Spontaneous alternation was evaluated by scoring the order of entries into each arm during the 5 min of the test trial. Spontaneous arm alternation (%) was defined as: number of arm alternations/(total number of arm visits-2) × 100 Spontaneous arm alternation (%) was defined as: number of arm alternations/(total number of arm visits-2) × 100

#### Novel object recognition:

The test was performed in two consecutive days. On day one, mice were placed in the center of an empty open box and allowed to explore for 5 minutes. The box was cleaned with 70% ethanol between trials. On day 2, the mice were placed back to an open box with two identical objects in the center and allowed to explore for 5 minutes. Thirty minutes later, mice were exposed again to a familiar and a novel object and allowed to explore for 5 minutes. The exploring activity (facing, touching or sniffing the object) was monitored and analyzed using AnyMaze in a double-blinded manner, and the percent of the time spent exploring the novel vs. familiar objects was calculated.

### Data analysis

Statistical analysis was performed using GraphPad Prism 10 (GraphPad Software, Inc). Histological, cerebrovascular, and behavioral analyses were conducted in a blinded fashion. Animals were randomly assigned to experimental groups. No animals were excluded. The number of mice required for assessing statistical significance of pre-specified effects was estimated by power analysis based on preliminary results and previous experience with the models used in the lab. Two-group comparisons were analyzed by the two-tailed t-test. Multiple comparisons were evaluated by one-way or two-way analysis of variance and Tukey’s test. Differences were considered statistically significant for probability values less than 0.05. Data are expressed as means ± SEM.

## Data Availability

The authors declare that the data supporting the findings of this study are available within the article and Extended Data Figures.
